# Viabahn endoprosthesis for femoropopliteal aneurysm repair: safety, success rates, and long-term patency

**DOI:** 10.1186/s42155-024-00465-3

**Published:** 2024-07-08

**Authors:** Jan M. Brendel, Tobias Mangold, Markus Pfändler, Benedikt Stenzl, Mateja Andic, Jonas Mück, Jörg Schmehl, Patrick Krumm, Christoph Artzner, Gerd Grözinger, Arne Estler

**Affiliations:** 1 Department of Radiology, Diagnostic and Interventional Radiology, University of Tübingen, Hoppe-Seyler-Straße 3, 72076 Tübingen, Germany; 2 Department of Anesthesiology and Intensive Care Medicine, Tübingen University Hospital, Tübingen, Germany; 3 Department of Thoracic and Cardiovascular Surgery, Tübingen University Hospital, Tübingen, Germany; 4 Department of Radiology, Diakonie Klinikum, Stuttgart, Germany

**Keywords:** Viabahn, Endoprosthesis, Stent Graft, Aneurysm, Endovascular, Repair

## Abstract

**Background:**

The Viabahn endoprosthesis has become a vital option for endovascular therapy, yet there is limited long-term data on its effectiveness for peripheral aneurysm repair. This study aimed to evaluate the safety, technical and clinical success, and long-term patency of the Viabahn endoprosthesis for treating femoropopliteal aneurysms.

**Methods:**

This retrospective tertiary single-center study analyzed patients who underwent a Viabahn endoprosthesis procedure for femoropopliteal aneurysm repair from 2010 to 2020. Intraoperative complications, technical and clinical success rates, and major adverse events (MAE, including acute thrombotic occlusion, major amputation, myocardial infarction, and device- or procedure-related death) at 30 days were assessed. Incidence of clinically-driven target lesion revascularisation (cdTLR) was noted. Patency rates were evaluated by Kaplan–Meier analysis.

**Results:**

Among 19 patients (mean age, 72 ± 12 years; 18 male, 1 female) who underwent aneurysm repair using the Viabahn endoprosthesis, there were no intraoperative adverse events, with 100% technical and clinical success rates. At the 30-day mark, all patients (19/19, 100%) were free of MAE. The median follow-up duration was 1,009 days [IQR, 462–1,466]. Popliteal stent graft occlusion occurred in 2/19 patients (10.5%) after 27 and 45 months, respectively. Consequently, the primary patency rates were 100%, 90%, 74% at 12, 24, and 36–72 months, respectively. Endovascular cdTLR was successful in both cases, resulting in sustained secondary patency at 100%.

**Conclusion:**

The use of Viabahn endoprostheses for femoropopliteal aneurysm repair demonstrated technical and clinical success rates of 100%, a 0% 30-day MAE rate, and excellent long-term patency.

## Introduction

The Viabahn endoprosthesis is a heparin-bonded stent graft that serves as an endovascular treatment option for peripheral artery disease [1–3], and has been expanded to include peripheral aneurysm repair [4]. Peripheral aneurysms, encompassing femoral and popliteal aneurysms, are less common than their abdominal aortic counterparts [5–8]. They typically affect men over 60 years of age and often occur alongside a variety of cardiovascular risk factors and comorbidities [9]. The potential consequences of femoropopliteal aneurysms are diverse, ranging from thrombosis and distal embolization to acute ischemia and compression of adjacent structures, with instances of rupture and limb loss [10–14]. In order to prevent unfavorable outcomes, it is imperative to provide optimal treatment. Elective open surgical repair via interposition or bypass graft is considered the gold standard [6, 15, 16]. Given the predominantly elderly patient demographic and the complexity of associated risks, endovascular treatment is frequently necessary due to its minimally invasive nature. The Viabahn endoprosthesis is widely recognized for its precise delivery capability and structural stability. However, despite its growing use in aneurysm therapy, there remains limited comprehensive data on its long-term performance on femoropopliteal aneurysm repair. Long-term outcome data is of significant importance in order to provide evidence-based treatment decisions, particularly in patients with a long enough life expectancy to suffer from potential long-term adverse events such as graft occlusion. Therefore, it is crucial to obtain robust performance data on safety and long-term patency, especially in a real-world setting [17, 18].

Consequently, the purpose of this investigation is to assess the safety, technical and clinical success, as well as the long-term outcomes of the Viabahn endoprosthesis for the management of femoropopliteal aneurysms.

## Methods

### Patient population

In this tertiary single-center study (Tuebingen, Germany), adult patients (age 18 years or older) who underwent femoropopliteal aneurysm repair using the Viabahn endoprosthesis between July 2010 and October 2020 were consecutively recruited and retrospectively analyzed. Endovascular treatment was indicated following the decision of an interdisciplinary tumor board for symptomatic aneurysms or for aneurysms with a diameter of ≥ 25 mm in femoral aneurysms and ≥ 20 mm in popliteal aneurysms [6, 16]. An all-comers approach was used. Cardiovascular risk factors were comprehensively documented, encompassing dyslipidemia, family history of premature coronary artery disease, hypertension, non-insulin-dependent (NIDDM) or insulin-dependent (IDDM) diabetes mellitus, obesity, and smoking status categorized as current, former, or never smoked. Comorbidities were recorded, renal function was assessed by measuring the glomerular filtration rate (GFR), and chronic renal failure (GFR < 60 mL/min/1.73m^2^) or current hemodialysis were noted. Evidence of bacteremia and known uncorrectable hypercoagulability were documented. The study received approval from the institutional review board (approval number: 254/2021BO1, date: 2021/08/20) and complied with the regulations of the Health Insurance Portability and Accountability Act (HIPAA), the need for informed consent was waived.

### Study procedure

All procedures were conducted under local anesthesia or conscious sedation. Following the establishment of vascular access, an angiographic catheter was navigated to the distal aspect of the target artery with the assistance of a guidewire. Subsequently, angiography of the target artery was conducted for aneurysm identification. The guidewire was replaced with a stiff wire, and the sheath was substituted with a dedicated 6F to 12F sheath. The selection of stent graft size was tailored to each patient based on pre-procedural imaging and/or intraoperative measurements. The diameter and length of the stent graft were chosen to ensure optimal apposition to the vessel wall and adequate coverage of the aneurysm. The sheath was advanced either to the distal side or as close as possible to the intended target artery. To deliver the stent graft, a stiff 0.018- or 0.035-inch wire was used. The Viabahn endoprosthesis, with diameters ranging from 5 to 13 mm and lengths ranging from 2.5 to 25.0 cm, was inserted and positioned over the intended target artery site for deployment. The characteristics of the implantation procedure were evaluated, including the number of stents employed and the dimensions (diameter in mm and length in cm) of the endoprostheses. Following the deployment of the device, immediate angiography was conducted to confirm the complete elimination of the aneurysm. Heparin (5,000 IU) was administered for the purpose of anticoagulation. The vascular access site was subsequently closed using a dedicated vascular closure device (for sheaths ≥ 6F). For sheaths exceeding 8F, suture-mediated closure devices were used for preclosing. Following the procedure, anticoagulation therapy was advised, with the recommendation of dual antiplatelet therapy, comprising daily doses of aspirin (100 mg) and clopidogrel (75 mg), to be administered for a minimum of six months, in order to reduce the risk of stent graft thrombosis. Consequently, a single dose of aspirin (100 mg per day) was prescribed as lifelong antiplatelet therapy.

### Technical and clinical success rates and follow-up

The acute technical success rate was assessed according to predetermined criteria, which included the successful placement of the Viabahn stent graft, the complete elimination of perfusion of the aneurysm sac, and the preservation of blood flow in the artery, as verified by final target site angiography. The clinical success criterion was the sustained blood flow through the repaired vessel for 30 days. Prior to hospital discharge, all patients underwent a CT angiography or ultrasound evaluation to confirm adequate blood flow. A follow-up care protocol was advised subsequent to the index procedure. Physical examination and duplex ultrasound visits were scheduled at regular 30-day, 3-month, 6-month, and 12-month intervals, with annual check-ups thereafter.

### Study endpoints

The primary safety endpoint of the study was the occurrence of major adverse events (MAE) within 30 days, including acute thrombotic occlusion, myocardial infarction, major limb amputation, and death. The secondary efficacy endpoint comprised the primary and secondary patency rates. In the event of stenosis or occlusion of the endoprosthesis, the time point of diagnosis after the initial procedure was documented, as well as the type of clinically driven target lesion revascularization (cdTLR) procedure, whether surgical or endovascular. The incidence of major or minor amputation was recorded.

### Statistical analysis

The normality of data was assessed by the Shapiro–Wilk test. For parametric data, continuous variables are expressed as the mean ± standard deviation, whereas non-parametric data are presented as the median [IQR]. Categorical data are provided as the numerator/denominator (percentage). The Kaplan–Meier method was used to estimate patency survival curves (SPSS version 29.0, IBM).

## Results

### Patient characteristics

A total of 19 patients underwent endovascular aneurysm repair utilizing the Viabahn endoprosthesis, comprising 18 men and 1 woman, with a mean age of 72 ± 12 years. A detailed account of the baseline characteristics of the patients is provided in Table 1. Arterial hypertension was the most prevalent cardiovascular risk factor, detected in 11 out of 19 patients (57.9%), followed by dyslipidemia (8/19, 42.1%), and current smoking (7/19, 36.8%). The most prevalent comorbidity was coronary artery disease, present in 7 out of 19 patients (36.8%), followed by chronic obstructive pulmonary disease in 4 out of 19 patients (21.1%). The mean GFR was 74 ± 25 mL/min/1.73m^2^. Chronic renal failure was observed in 4 out of 19 patients (21.1%), and none of the patients were undergoing hemodialysis. Notably, none of the patients tested positive for bacteremia, nor exhibited uncorrectable hypercoagulability.

**Table 1:** Patient characteristics

Characteristic	Total number of patients n = 19
Age	
at intervention, yrs	72 ± 12
range, yrs	53–94
< 60 yrs	4/19 (21.1)
60–70 yrs	3/19 (15.8)
> 70 yrs	12/19 (63.2)
Sex at birth	
Male	18/19 (94.7)
Female	1/19 (5.3)
Body mass index, kg/m2	25 ± 3
Cardiovascular risk factors	
Arterial hypertension	11/19 (57.9)
Diabetes	
No	16/19 (84.2)
NIDDM	3/19 (15.8)
IDDM	0/19 (0.0)
Dyslipidemia	8/19 (42.1)
Family history of CAD	4/19 (21.1)
Obesity	2/19 (10.5)
Smoking status	
Current smoker	7/19 (36.8)
Former	4/19 (21.1)
Never	8/19 (42.1)
Comorbidities	
COPD	4/19 (21.1)
Cerebrovascular artery disease	1/19 (5.3)
Coronary artery disease	7/19 (36.8)
Atrial fibrillation	2/19 (10.5)
Renal function	
GFR (mL/min/1.73m2)	74 ± 25
Chronic renal failure^a^	4/19 (21.1)
Hemodialysis	0/19 (0.0)
Evidence of bacteremia	0/19 (0.0)
Uncorrectable hypercoagulability	0/19 (0.0)

Values are mean ± SD, range, or numerator/denominator (%)

*CA*D coronary artery disease, *COPD* chronic obstructive pulmonary disease, *GFR* glomerular filtration rate, *IDDM* insulin-dependent diabetes mellitus, *NIDDM* non-insulin-dependent diabetes mellitus

^a^GFR < 60 mL/min/1.73m^2^

### Procedural characteristics

**Fig. 1:**
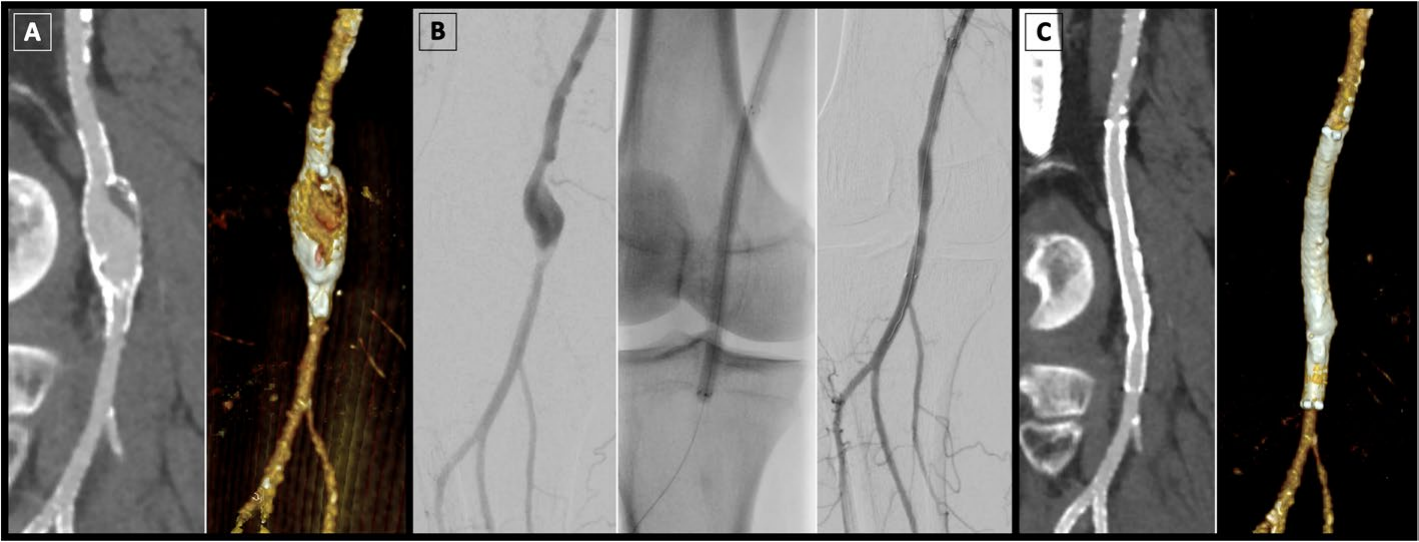
Viabahn endoprosthesis for endovascular treatment of popliteal aneurysm. Example of a 61-year-old man with peripheral artery disease and moderate claudication. **A** CT angiography (CTA) revealed a popliteal aneurysm and stent fracture in the P2 segment. **B** Subsequently, the patient underwent Viabahn stent graft procedure. Final target site angiography demonstrated successful aneurysm exclusion by the implanted Viabahn with preserved blood flow and no peripheral complications. **C** Follow-up CTA at 2 years demonstrated sustained blood flow through the device

**Fig. 2:**
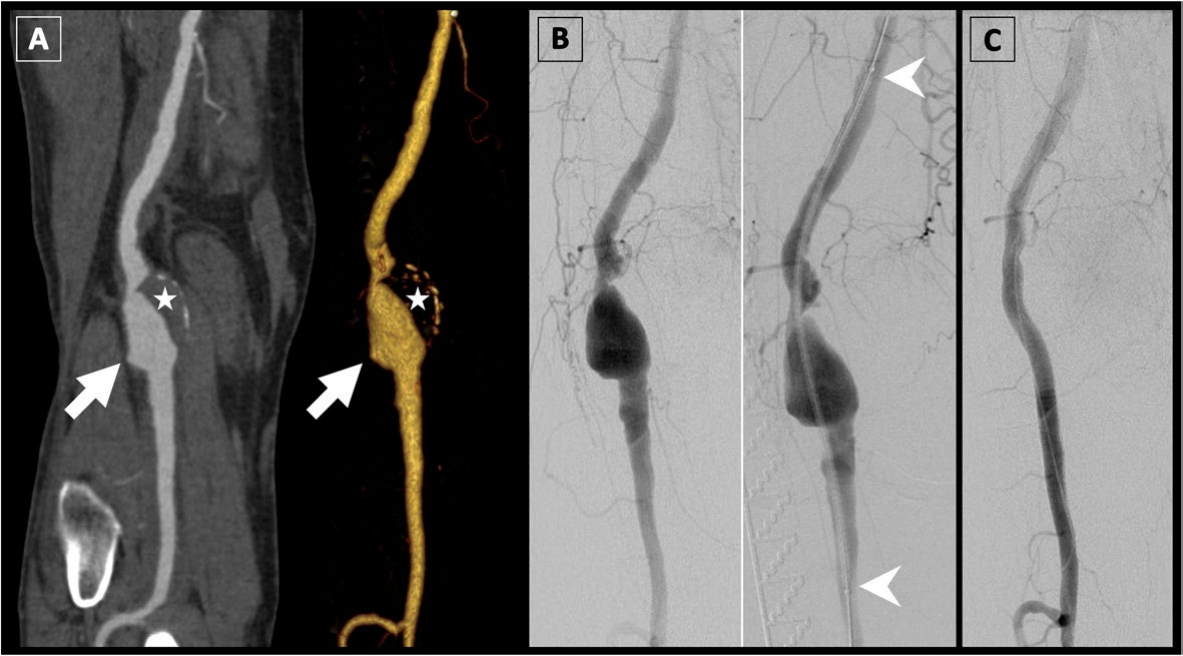
Viabahn endoprosthesis for popliteal aneurysm repair. A 73-year-old man was referred for **(A)** CT angiography, which revealed a popliteal aneurysm (arrows) with substantial lumen thrombus (stars). **B** Stent graft repair was performed using a Viabahn endoprosthesis (proximal and distal ends of the stent graft indicated by arrowheads). **C** Final target site angiography confirmed successful exclusion of the aneurysm and preservation of blood flow

**Fig. 3:**
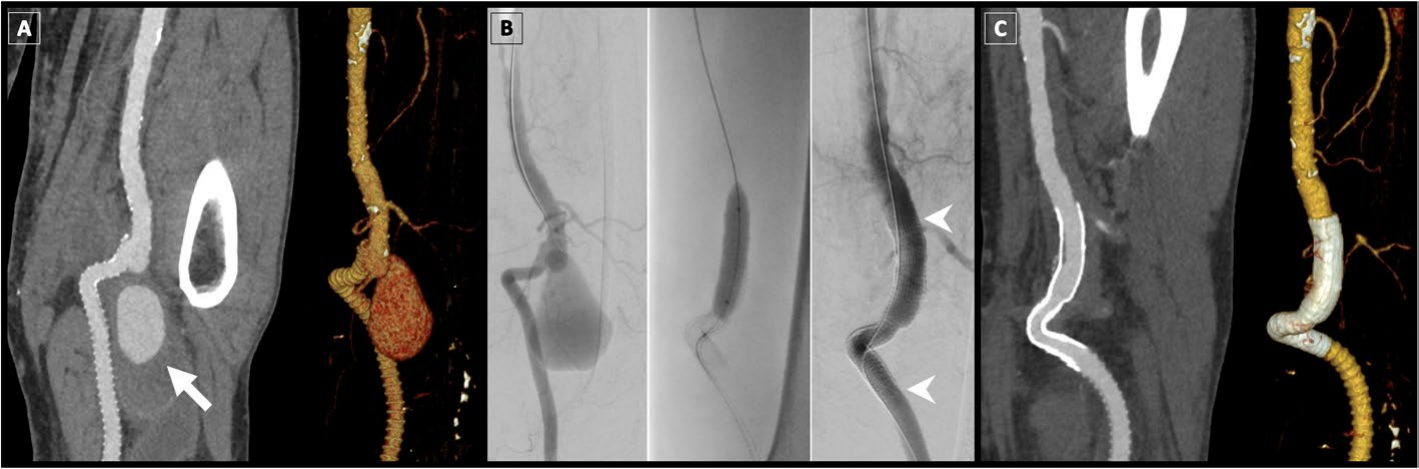
Viabahn endoprosthesis for femoral aneurysm repair. A 77-year-old man with previous P1/P3 bypass operation presented with swelling and pain in the distal posterior aspect of his thigh.** A** CT angiography (CTA) revealed the presence of a false aneurysm of the distal superficial femoral artery (arrow). **B** The patient underwent stent graft repair using a Viabahn endoprosthesis (indicated by arrowheads). The successful exclusion of the aneurysm with preservation of blood flow was confirmed by final target site angiography. **C** Follow-up CTA at 1 year demonstrated sustained preservation of blood flow

Aneurysms were targeted in the popliteal artery in 16 out of 19 patients (84.2%), and in the superficial femoral artery in 3 out of 19 patients (15.8%), see Figs. 1, 2, 3 for exemplary cases. Regarding aneurysm morphology, 16/19 cases (84.2%) were fusiform aneurysms, 2/19 cases (10.5%) were saccular aneurysms, and 1/19 cases (5.3%) was an eccentric aneurysm. Aneurysms with thrombus occupying more than 50% of the lumen were recorded in 9/19 patients (47.4%). A tibial runoff of less than 2 vessels was observed in 5 out of the 19 patients (26.3%). A total of 26 endoprostheses were placed, with the most frequently employed device diameter measuring 6 mm (13/26, 50.0%), followed by > 8 mm (7/26, 26.9%), 8 mm and 7 mm (each 3/26, 11.5%), as shown in Table 2. The most commonly utilized stent graft length was 10.0 cm (10/26, 38.5%), followed by 15.0 cm and 5.0 cm (each 5/26, 19.2%). Devices measuring 25.0 cm were utilized in four out of 26 cases (15.4%), 7.5 cm and 2.5 cm were used in each one out of 26 cases (3.8%). There were no intraoperative adverse events, and technical success was achieved in all 19/19 procedures (100%).

**Table 2:** Procedural characteristics

Characteristic	Total number of patients n = 19
	Total number of deployed devices n = 26
Target Aneurysm	
Superficial femoral artery	3/19 (15.8)
Popliteal artery	16/19 (84.2)
Aneurysm shape	
Fusiform	16/19 (84.2)
Eccentric	1/19 (5.3)
Saccular	2/19 (10.5)
Aneurysm metrics	
Length (mm)^a^	49 [30–60]
Diameter (mm)^a^	16 [13–20]
Neck length (mm)^b^	10 [6–13]
Height (mm)^b^	45 [24–65]
Width (mm)^b^	34 [23–44]
Aneurysm thrombus > 50% lumen	9/19 (47.4)
Proximal vessel diameter (mm)	7 [6–9]
Distal vessel diameter (mm)	6 [6–7]
Tibial runoff	
≥ 2-vessel runoff	14/19 (73.7)
< 2-vessel runoff	5/19 (26.3)
Number of implanted devices	
1	12/19 (63.2)
2	7/19 (36.8)
3	0/19 (0.0)
Device diameter (n, % of 26 deployed)	
5 mm	0/26 (0.0)
6 mm	13/26 (50.0)
7 mm	3/26 (11.5)
8 mm	3/26 (11.5)
> 8 mm	7/26 (26.9)
Device length (n, % of 26 deployed)	
2.5 cm	1/26 (3.8)
5.0 cm	5/26 (19.2)
7.5 cm	1/26 (3.8)
10.0 cm	10/26 (38.5)
15.0 cm	5/26 (19.2)
25.0 cm	4/26 (15.4)
Proximal landing zone (mm)	25 [18–32]
Distal landing zone (mm)	21 [16–28]
Intraoperative adverse events	0/19 (0.0)
Technical success^c^	19/19 (100)

Values represent frequencies as numerator/denominator (%), or median [IQR]

^a^Metrics for fusiform and eccentric aneurysms

^b^Metrics for saccular aneurysms

^c^Technical success was defined as the complete deployment of the device with total elimination of perfusion of the aneurysm sac, and the preservation of blood flow in the artery, as determined by final target site angiography

### Clinical success and major adverse events within 30 days

All 19/19 procedures (100%) demonstrated clinical success, as shown in Table 3. There were no device- or procedure-related deaths, myocardial infarctions, major amputations, or acute thrombotic occlusions within 30 days of stent graft procedure (0/19 patients, 0.0%). Thus, all patients (19/19, 100%) were free from any major adverse events at the 30-day mark.

**Table 3:** Clinical and procedural outcomes

Parameter	Total number of patients n = 19
Clinical success^a^	19/19 (100)
MAE within 30 days	
Death^b^	0/19 (0.0)
Myocardial infarction	0/19 (0.0)
Major amputation	0/19 (0.0)
Acute thrombotic occlusion	0/19 (0.0)
Follow-up duration (days)	1009 [462–1466]
Follow-up duration range (days)	34–2806
cdTLR	
Total	2/19 (10.5)
Endovascular revascularization	2/19 (10.5)
Surgical treatment	0/19 (0.0)
Major amputation	1/19 (5.3)
Minor amputation	0/19 (0.0)

Values are frequencies as numerator/denominator (%), median [IQR], or range

*MAE* major adverse event, *cdTLR* clinically driven target lesion revascularization

^a^The clinical success criterion was defined as the sustained blood flow through the repaired vessel within 30 days. ^b^device- or procedure-related

### Long-term outcome

**Fig. 4:**
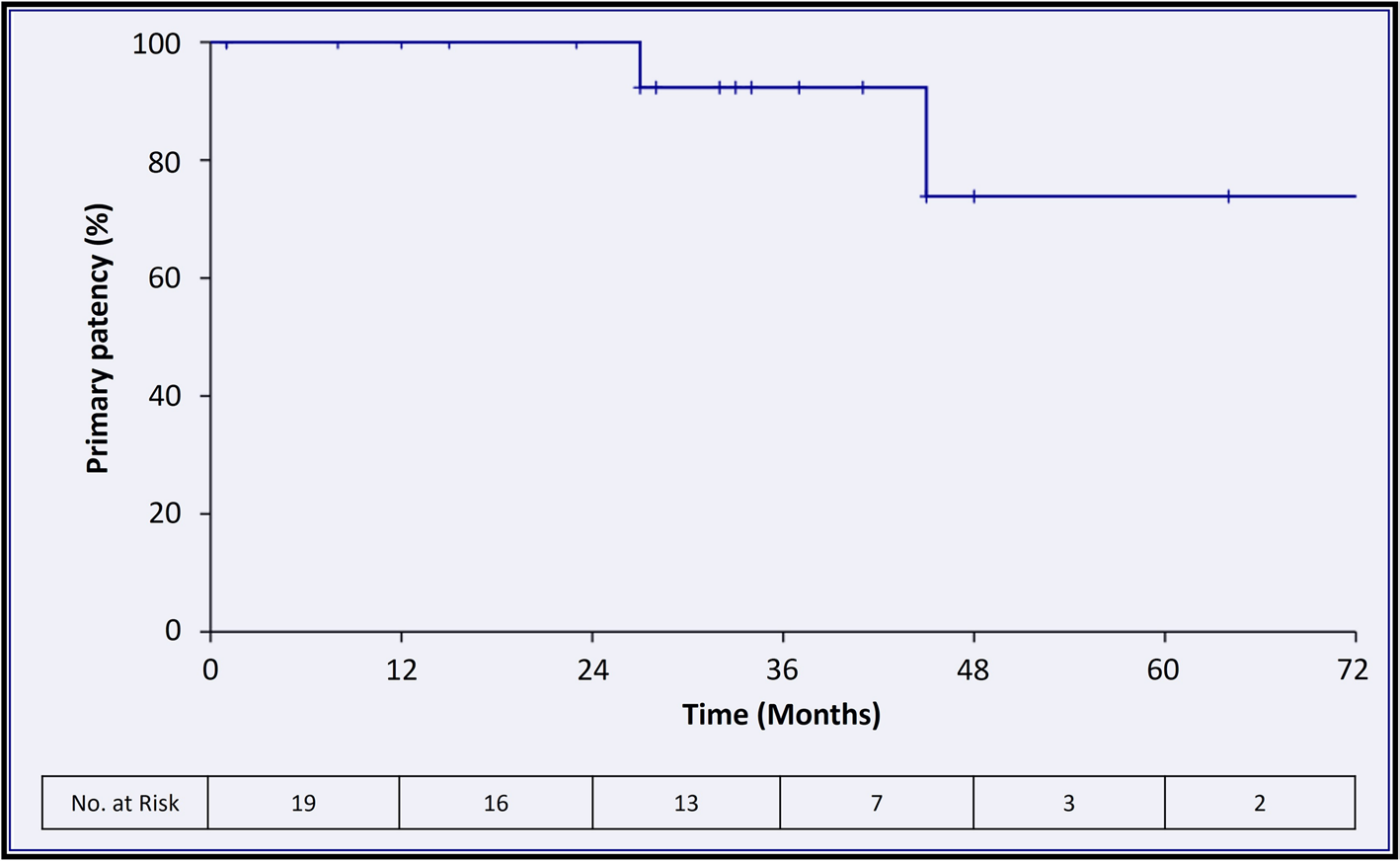
Long-term primary patency of the Viabahn endoprosthesis for femoropopliteal aneurysm management. The curve shows the primary patency (%) of the Viabahn endoprosthesis for the treatment of femoropopliteal aneurysms over a period of 72 months following the procedure

The median follow-up duration was 1,009 days [IQR, 462–1,466], with a range of 34 to 2,806 days. Two cases of popliteal stent graft occlusion were observed. One graft occlusion of a 6 mm Viabahn device occurred at 27 months post-procedure in a 58-year-old male patient with arterial hypertension, dyslipidemia, current smoking, and a family history of premature coronary artery disease. Another graft occlusion of a 10 mm Viabahn device occurred at 45 months post-procedure in a 75-year-old male patient with non-insulin dependent diabetes mellitus, chronic renal failure, a family history of premature coronary artery disease, and a two-vessel run-off. In both instances, endovascular revascularization was successful. The patient with popliteal occlusion at 27 months had pre-existing stage IV (Fontaine) peripheral artery disease, manifested by poor distal run-off (collateral vessels only). Despite the successful endovascular revascularization of the stent graft, lower leg amputation was subsequently considered the ultimate treatment option. Kaplan–Meier analysis unveiled primary patency rates of 100%, 90%, and 74% at 12, 24, and 36–72 months post-endoprosthesis procedure, respectively, see Fig. 4. Secondary patency was observed to be sustained at 100%.

## Discussion

This study comprehensively evaluated the performance of the Viabahn endoprosthesis for femoropopliteal aneurysm management. The device demonstrated a good safety and success profile, with no intraoperative adverse events, technical and clinical success rates reaching 100%, and a 30-day major adverse event rate of 0%. Long-term outcomes were favorable, with primary patency rates of 100%, 90%, and 74% at 12, 24, and 36–72 months, respectively. Secondary patency was sustained at 100%.

The high technical success rate can be attributed to the excellent maneuverability facilitated by the high flexibility of the device when navigating complex arterial structures. The absence of intraoperative complications further substantiates the safety profile of the device. The 30-day major adverse event rate of 0% observed in our study provides valuable evidence supporting the device's safety across various pathologies, including stenosis, vessel injury, and aneurysms [16, 19–24]. Our results also showed sustained efficacy in the long-term, with primary patency rates of 100%, 90%, and 74% at 12, 24, and 36–72 months post initial implantation, respectively. The gradual modest decrease in primary patency rates over the long-term is consistent with previously reported reported primary patency rates at 60 months of 69%, confirming the long-term durability of Viabahn stents in the treatment of peripheral aneurysms [4]. This may be attributed, in part, to the device's capacity to withstand compressional stress during limb movement without fracture of the prosthesis, which is of critical importance for ensuring adequate perfusion distally [25]. In accordance with previous studies, we observed a high secondary patency, thereby reinforcing the concept that the Viabahn is an effective conduit for revascularization procedures [4, 26]. However, several factors can influence the patency of stent grafts, including cardiovascular risk factors, the size of the stent graft, the effectiveness of anticoagulation therapy, the presence of peripheral artery disease, and distal runoff. Each of these factors plays a critical role in determining the long-term success of the procedure and should be carefully considered during patient evaluation and treatment planning. In both instances of stent graft occlusion observed in our study, the patients exhibited cardiovascular risk factors and impaired distal run-off. Additionally, one patient had a smaller device diameter of 6 mm, which had previously been demonstrated to be associated with a reduced primary patency [27]. The findings of our study, including technical and clinical success as well as patency, demonstrate the efficacy of endovascular strategies, particularly for patient populations with underlying health conditions or complex anatomy requiring minimally invasive treatment. The advantages of endovascular aneurysm repair include a lower incidence of wound complications and a shorter hospital stay compared to open surgical treatment [15, 16]. Nevertheless, open repair remains an option in aneurysm therapy, shown to carry a lower risk of primary patency loss within the initial three years post-procedure; however, secondary patency rates are comparable between open and endovascular treatments for popliteal aneurysm treatment [15, 16]. With regard to the inherent limitations of open surgery [28], including the risk of access-related injuries and prolonged healing, the endovascular aneurysm management represents a viable alternative, offering durable arterial repair [4, 16]. Taken together, choosing the most appropriate approach requires a comprehensive assessment of lesion characteristics, patient-related factors, and treatment objectives to minimize procedural risks. This requires adopting a collaborative, interdisciplinary framework for decision-making that incorporates the expertise of both open surgical and interventional specialists, ultimately aiming to advance patient care.

This study has certain limitations that warrant consideration. First, it employs a retrospective, single-arm analysis design and lacks a control group, thereby limiting the ability to draw direct comparative conclusions. Second, the examination is limited to a patient population from a single tertiary center, which constrains the generalizability of our findings to other centers and countries. Third, the selected real-world approach, while inclusive, may pose challenges in the identification and management of confounding variables.

## Conclusions

The use of the Viabahn endoprosthesis for femoropopliteal aneurysm repair had 100% technical and clinical success rates, a 0% major adverse event rate within 30 days, and yielded excellent long-term primary and secondary patency. Further studies are needed to fully evaluate the factors that influence long-term performance in order to further refine the profiling of patients who derive optimal benefits from endovascular repair over open surgical repair.

## Data Availability

The datasets used and/or analyzed during the current study are available from the corresponding author on reasonable request.

## Abbreviations

cdTLR: Clinically driven target lesion revascularization
IDDM: Insulin-dependent diabetes mellitus
MAE: Major adverse events
